# A deep learning based encoder-decoder model for speed planning of autonomous electric truck platoons

**DOI:** 10.1016/j.heliyon.2024.e31836

**Published:** 2024-05-31

**Authors:** S. Karthik, G. Rohith, K.B. Devika, Shankar C. Subramanian

**Affiliations:** a Department of Mechanical Engineering, Indian Institute of Technology Madras, Chennai 600036, India; b Orbital Astronautics, Thames Court, Goring, Oxfordshire, RG8 9AQ, United Kingdom; c Faculty of Environment, Science and Economy, University of Exeter, Exeter, EX4 4QF, United Kingdom; d Department of Engineering Design, Indian Institute of Technology Madras, Chennai 600036, India

**Keywords:** Deep learning, Drive cycle, Electric trucks, Encoder-decoder model, Platoon, State-of-charge

## Abstract

Electric truck platooning offers a promising solution to extend the range of electric vehicles during long-haul operations. However, optimizing the platoon speed to ensure efficient energy utilization remains a critical challenge. The existing research on implementing data-driven solutions for truck platooning remains limited and implementing first principles solution is still a challenge. However, recognizing the resemblance of truck platoon data to a time series serves as a compelling motivation to explore suitable analytical techniques to address the problem. This paper presents a novel deep learning approach using a sequence-to-sequence encoder-decoder model to obtain the speed profile to be followed by an autonomous electric truck platoon considering various constraints such as the available state of charge (SOC) in the batteries along with other vehicles and road conditions while ensuring that the platoon is string stable. To ensure that the framework is suitable for long-haul highway operation, the model has been trained using various known highway drive cycles. Encoder-decoder models were trained and hyperparameter tuning was performed for the same. Finally, the most suitable model has been chosen for the application. For testing the entire framework, drive cycle/speed prediction corresponding to different desired SOC profiles has been presented. A case study showing the relevance of the proposed framework in predicting the drive cycle on various routes and its impact on taking critical policy decisions during the planning of electric truck platoons has also been presented. This study would help to efficiently plan the feasible routes for electric trucks considering multiple constraints such as battery capacity, expected discharge rate, charging infrastructure availability, route length/travel time, and other on-road operating conditions while also maintaining stability.

## 1 Introduction

Despite diesel-powered heavy trucks being significant contributors to greenhouse gas emissions, the electrification of trucks is still in its early stages due to various challenges. Major obstacles include the higher cost and weight of batteries, as well as the lack of charging infrastructure along long routes. These factors hinder the realization of truck electrification for long-haul freight transportation [1], [2], [3]. In a truck platoon formation, the trucks travel one behind the other in closed formations leading to reduced aerodynamic drag force on each vehicle. Since during high-speed cruising of heavy trucks, more amount of energy is consumed to overcome the drag force, a closely spaced platoon formation could potentially lead to a significant reduction in energy consumption [4]. Hence, adopting platoon formation for long-haul battery electric truck operation would lead to reduced usage of energy from the batteries and hence improved driving range [5].

An extensive amount of literature is available in the field of energy-optimal and stable platoon formation of conventional diesel engine-powered heavy trucks [6]. Controller-based approaches were undertaken in [7], [8] to solve this problem. The drayage problem in platoons is discussed in [9], [10] and they attempted to develop efficient algorithms for solving the same. However, only a few dealt with platooning of electric vehicles, particularly electric trucks [11]. Eco-driving advisory strategies for heterogeneous platoon consisting of gasoline vehicles and electric vehicles have been presented in [12]. Lee et al. reported a model-based optimization technique to determine the optimal number and configuration of electric vehicle platoons [13]. An optimization approach to schedule electric commercial vehicle platoon formation is presented in [14]. Devika et al. performed a stability analysis of a battery electric truck platoon for various on-road conditions [15]. To the best of the authors' knowledge, speed planning of electric truck platoon formation for better battery utilization and range extension has not been adequately addressed yet.

During an electric truck operation, the electric power demanded by the motor depends upon various factors such as vehicle speed, tire-road interface, vehicle load, and intervehicular spacing between the trucks (in platoon formation). Depending upon the operating conditions and battery state of charge (SOC) in each truck, the range that could be covered by an electric truck platoon varies. One possible way to cover the required range (to complete a particular route) is to adjust the platoon speed based on the operating conditions that exist in that route. In this regard, the goal here is to plan the desired platoon speed a priori such that the required range is completed utilizing the available SOC in the batteries or the platoon reaches the next available charging hub along the route. However, the dependency between platoon speed and energy consumption cannot be easily captured by the first principles approach since it depends upon various route and vehicle parameters such as road condition (characterized by tire road friction coefficient), and mass of the vehicle. Moreover, this also depends on the time-headway magnitude (that characterizes the intervehicular spacing between the trucks), which is to be maintained in the platoon. Hence, a data-driven approach has been adopted to predict the desired platoon speed while capturing all the aforementioned conditions and considering available battery SOC.

Deep learning has emerged as a powerful technique for solving complex problems in various domains such as computer vision, natural language processing, speech recognition, and robotics [16]. It has also contributed to advancements in the medical industry in applications pertaining to protein-protein interactions, drug-disease interactions, and protein-disease interactions [17], [18]. Deep learning has recently found applications in the electric vehicle domain such as the speed profile prediction of plug-in hybrid electric buses for improved energy economy [19] and prediction of battery capacity [20]. Recurrent neural networks (RNNs), a deep learning approach, are a class of neural networks that can handle sequential data such as time-series, text, and speech. However, traditional RNNs suffer from the vanishing gradient problem, which makes it difficult for them to learn long-term dependencies in sequential data. Long Short-Term Memory (LSTM) networks were proposed as a solution to this problem, allowing RNNs to capture long-term dependencies in sequential data. LSTMs use a gating mechanism to control the flow of information and overcome the vanishing gradient problem.

Encoder-decoder LSTM models are a type of RNN architecture that has been widely used in natural language processing, speech recognition, machine translation, and other sequence-to-sequence learning tasks [21], [22]. The basic idea behind these models is to encode an input sequence into a fixed-length vector representation, which is then decoded by another LSTM network to generate the output sequence. This approach allows the model to handle input sequences of variable length and to generate output sequences of arbitrary length. The encoder-decoder LSTM model has shown remarkable success in several real-world applications, making it an important topic of research in the field of deep learning. In the case of truck platooning, both energy and speed can be represented as time series data, which is similar to a case of language translation where both the input and the output are of similar format. The parallelism between these two use-cases serves as a key factor in choosing this model architecture. In this context, an encoder-decoder LSTM model has been developed to predict the speed profile of an electric truck platoon for any given SOC profile under different road conditions, vehicle masses, and time headway magnitudes.

Since SOC is a cumulative profile and it is better to have independent data points to develop the model, it has been converted to an instantaneous power consumption profile before passing it to the LSTM model as input. In the case of any Deep Learning model, the training data should be true data collected from various real-life sources (i.e., a collection of known mapped translations). The ideal scenario for this case is to physically run the truck platoon with known configurations and measure all the required parameters which is difficult and can be a disadvantage to this approach. However, the LSTM model has been trained using the data collected from an electric truck platoon framework, developed in Matlab Simulink®, which encompasses critical features such as complete longitudinal vehicle dynamics with resistive forces, wheel dynamics, tire model, brake model, electric motor model, and battery model in the platoon framework. Since the model is extensive and it considers vehicle-dynamic factors to the tire level, it can be said that the model is realistic and reliable to be used. For collecting training data set using the above, known highway drive cycles were used and power profiles were generated under different operating conditions.

The planned drive cycle/speed from the framework could potentially be used to plan the journey with respect to the initial available SOC in the trucks, battery capacity, and the locations of the charging stations. On predicting the drive cycles for any particular route, the presented approach enables to find out the kinematic parameters [23] associated with the drive cycle to be followed by the electric truck platoon. These parameters could give more insights about the route, and help to take decisions on charging location placement, battery capacity, and further design strategies.

**Figure 1:**
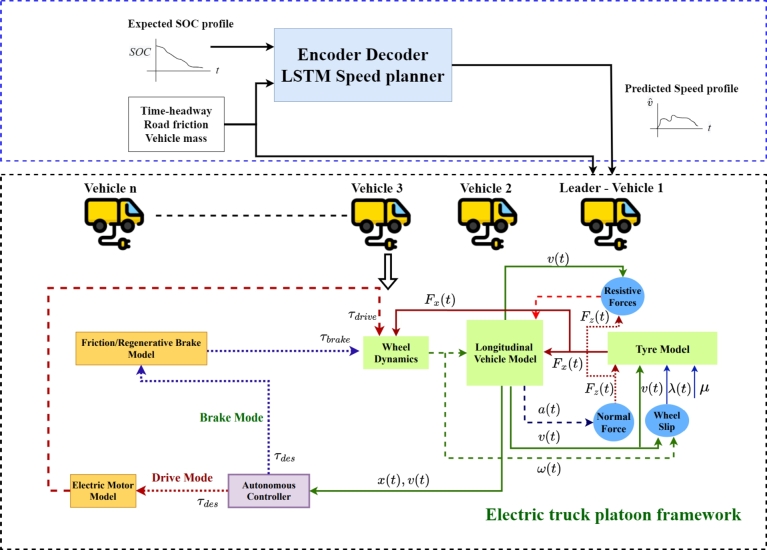
Proposed encoder-decoder LSTM speed planner using autonomous electric truck platoon framework. All the symbols and notations are defined in Section 2.

Based on the discussions above, the major contributions of this paper are:

• Design of an autonomous string stable electric truck platoon framework.
• Design of an encoder-decoder LSTM model that predicts the speed profile/drive cycle to be followed by a platoon for meeting the battery energy requirements.
• The framework has been developed such that the predicted drive cycle ensures a string stable operation in different operating conditions.
• The presented speed planner could be used to derive kinematic parameters associated with drive cycles, which helps to take policy decisions on the electric truck routing problem.
  The layout of this overall speed planner is shown in Fig. 1. All the symbols and notations are described in the following section.

## 2 Electric truck platoon framework

A framework for autonomous electric truck platoon has been developed for synthesizing the data set for developing the model. The framework has been developed based on the following assumptions:

• Solely the longitudinal movement of the truck is taken into account.
• The parameters of both the trucks and their batteries are available.
• All the trucks are assumed to have the same initial SOC during the start of a route and are equipped with battery packs of the same capacity.
• A Constant Time Headway (CTH) policy has been used to ensure a safe gap between the vehicles.

Each component of the platoon framework is explained below.

### 2.1 Vehicle dynamics model

Within the platoon formation, trucks are defined by employing the subsequent vehicle dynamics model. The dynamics of position and speed for the $i^{th}$ truck in the platoon are expressed in equation (1) as follows

(1) $$\overset{˙}{x_{i}}(t)=v_{i}(t),\overset{˙}{v_{i}}(t)=\Gamma (v_{i}(t),\tau_{i}(t)),$$

where, there are i=1,2,...,N trucks in the platoon, $v_{i}(t)$ and $\tau_{i}(t)$ are the longitudinal speed and drive/brake torque of each truck. $\Gamma (v_{i}(t),\tau_{i}(t))$ is defined in equation (2) as follows

(2) $$\Gamma (v_{i}(t),\tau_{i}(t))=\frac{1}{m_{i}}(F_{xfi}(\lambda_{fi}(t))+F_{xri}(\lambda_{ri}(t))-F_{Ri}(t)).$$

where $m_{i}$ is the mass of each truck, $F_{xfi}(\lambda_{fi}(t))$ and $F_{xri}(\lambda_{ri}(t))$ express the longitudinal forces acting at the interface between the front and rear tires and the road, respectively, and $\lambda_{fi}(t)$ and $\lambda_{ri}(t)$ are the longitudinal slip ratios of front and rear wheels, respectively. The slip ratios are given in equation (3) as follows

(3) $$\lambda_{fi}(t)=\frac{v_{i}(t)-r_{i}\;\omega_{fi}(t)}{v_{i}(t)},\;\lambda_{ri}(t)=\frac{v_{i}(t)-r_{i}\;\omega_{ri}(t)}{v_{i}(t)},$$

where, $r_{i}$ is the tire radius.

Equation (4) gives the wheel dynamics as

(4) $${\overset{˙}{\omega }}_{fi}(t)=\frac{1}{I_{fi}}(\tau_{fi}(t)-r_{i}F_{xfi}(\lambda_{fi}(t))),{\overset{˙}{\omega }}_{ri}(t)=\frac{1}{I_{ri}}(\tau_{ri}(t)-r_{i}F_{xri}(\lambda_{ri}(t))),$$

where $\omega_{fi}(t)$ and $\omega_{ri}(t)$ denote the angular speeds of the front and rear wheels at time *t*, while $I_{fi}$ and $I_{ri}$ represent the moment of inertia of the front and rear wheels respectively. The transmitted torques to the front and rear wheels are indicated by $\tau_{fi}(t)$ and $\tau_{ri}(t)$ respectively. The longitudinal forces $F_{xfi}(\lambda_{fi}(t))$ and $F_{xri}(\lambda_{ri}(t))$ are determined using the Magic Formula (MF) tire model [24], which provides an accurate representation of tire-road interface forces across a wide range of operating conditions, making it suitable for this study. The resistive forces are given in equation (5) as

(5) $$F_{Ri}(t)=F_{ai}(t)+R_{xfi}(t)+R_{xri}(t)+F_{gradi}(t)=\rho \;a_{f}\;C_{D}\frac{v_{i}{\left(t\right)}^{2}}{2}+f_{r}(F_{zfi}(t)+F_{zri}(t))+m_{i}g\sin(\theta ),$$

where, $F_{ai}(t)$ represents drag, $R_{xfi}$ and $R_{xri}$ represent the front and rear rolling resistance forces respectively, and $F_{gradi}$ is the component of force caused due to the inclination of the road (*θ*). The aerodynamic drag force is given by $F_{ai}(t)=\rho \;a_{f}C_{D}\frac{v_{i}{\left(t\right)}^{2}}{2}$, where *ρ* is the air density, $C_{D}$ represents the aerodynamic drag coefficient, and $a_{f}$ represents the vehicle frontal area. The rolling resistance is the product of the rolling resistance coefficient, $f_{r}$, and the normal force at a tire-road interface [15]. The normal forces at the front and rear tire-road interface are given in equation (6) as

(6) $$F_{zfi}(t)=\frac{m_{i}gl_{r}\cos(\theta )-F_{ai}(t)h_{a}-m_{i}a(t)h_{cg}-m_{i}gh_{cg}\sin(\theta )}{L},F_{zri}(t)=\frac{m_{i}gl_{f}\cos(\theta )+F_{ai}(t)h_{a}+m_{i}a(t)h_{cg}+m_{i}gh_{cg}\sin(\theta )}{L}.$$

Here, a(t) is the longitudinal acceleration, $h_{cg}$ is the height of the centre of gravity (C.G.) of the vehicle, $h_{a}$ is the height of the location at which the equivalent aerodynamic force acts, *θ* represents the road inclination, and $l_{f}$ and $l_{r}$ are the longitudinal distance of the front axle and rear axle from the C.G. of the vehicle, and the wheelbase $L=l_{f}+l_{r}$.

### 2.2 Electric motor and pneumatic brake models

The electric motor can be represented as a transfer function (Equation (7)) [25],

(7) $$P_{m}(s)=m_{a}\frac{V_{dc}}{2}e^{\frac{-T_{dm}}{2}s}\frac{K_{m}}{\left(1+\tau_{m}s\right)},$$

where $m_{a}$ is the modulation index of the inverter circuit, $\frac{V_{dc}}{2}$ is the inverter gain, $T_{dm}$ is the PWM delay, $K_{m}$ and $\tau_{m}$ represent the electric motor's gain and time constant respectively. In this study, $m_{a}=0.9176$, $\frac{V_{dc}}{2}=400$ V, $T_{dm}=0.1$ ms, $K_{m}=218.7$, $\tau_{m}=0.05498$ ms [25]. The air brake system has been characterized using a Hardware in Loop [26] calibrated first order with delay transfer function model, and is given by (Equation (8))

(8) $$P(s)=\frac{\tau_{act}(s)}{\tau_{des}(s)}=\frac{1}{\left(1+\tau_{b}s\right)}e^{-T_{d}s},$$

where, $\tau_{des}$ and $\tau_{act}$ are the demanded and the actual brake torque developed, $T_{d}$ and $\tau_{b}$ denote time delay and time constant, respectively. The time constant ($\tau_{b}$) and time delay ($T_{d}$) have been empirically found to be $\tau_{d}=$ 260 ms and $T_{d}=$ 45 ms [26].

### 2.3 Braking strategy

A linear proportion has been considered in the distribution of braking forces between the front and rear axles (Equation (9)), such that,

(9) $$\frac{F_{bf}}{F_{br}}=\frac{\beta }{1-\beta },$$

where $F_{bf}$ and $F_{br}$ are the front and rear brake forces, respectively, and 0<β<1.

The braking strategy in electric vehicles can be represented as (Equation (10))

(10) $$F_{bf}=F_{bf_{fr}},F_{br}=F_{br_{fr}}+F_{br_{reg}},$$

$F_{bf}$ and $F_{br}$ represent the front and rear wheel braking forces, respectively, $F_{bf_{fr}}$ and $F_{br_{fr}}$ represent the friction brake force components of front and rear wheels, respectively, and $F_{br_{reg}}$ represents the regenerative braking force applied at the rear wheels, assuming rear wheel drive configuration. A parallel cooperative braking (PCB) strategy has been used in this study to characterize braking in the electric trucks in the platoon. The PCB strategy is given by Equation (11), which is as follows

(11) $$F_{br_{reg}}=\gamma \;F_{b},$$

where *γ* is the ratio of regenerative braking force to the total braking force.

### 2.4 Battery model

The required power for an electric truck, given the SOC of the battery pack is given by equation (12). It can be expressed as

(12) $$P_{bat}(SOC)=\frac{P_{mot}}{\eta_{m}}.$$

$P_{mot}=ma(t)v(t)+F_{R}(t)v(t)$ is the power required by the motor and $\eta_{m}$ denotes the efficiency of the motor.

The battery pack is presumed to consist of a combination of series and parallel cells ($N_{s}$ and $N_{p}$, respectively). Equation (13) quantifies the average power by each cell. It is given by

(13) $$P_{c}(SOC)=\frac{{\left(V(SOC)\right)}^{2}}{4R_{in}},$$

where, *V* is the cell voltage and $R_{in}$ is the cell internal resistance in ohms.

Equation (14) gives the number of cells needed to traverse a given distance. It is given as range (R) and can be expressed as follows

(14) $$N_{s}N_{p}=\frac{4P_{mot}R}{\eta_{m}{\left(V(SOC)\right)}^{2}}.$$

Equation (15) gives the number of cells in series, which can be determined by matching nominal battery pack and nominal motor voltages and assuming idle conditions. It is given by

(15) $$N_{s}=\lfloor \frac{V_{mot,nom}}{V_{c}}\rfloor .$$

$V_{c}$ and $V_{mot,nom}$ denote the voltage of an individual cell and the nominal motor voltage, respectively. ⌊.⌋ is the floor function. $N_{p}$ can be determined using the two previous equations.

### 2.5 Intervehicular spacing model

Equation (16) gives the gap between the $i^{th}$ and ${\left(i-1\right)}^{th}$ truck as

(16) $$d_{i}(t)=x_{i-1}(t)-x_{i}(t).$$

Equation (17) defines the inter-vehicular distance as

(17) $$s_{d}(t)=s_{o}+h_{i}v_{i}(t),$$

where $s_{o}$ and $h_{i}$ denote standstill spacing and the time-headway respectively. This study employs a non-variable time headway approach. Equation (18) denotes the spacing error in this case, which is described as

(18) $$e_{i}(t)=d_{i}(t)-s_{d}(t).$$

In order to ensure string stability [4], the spacing error tends to zero in all cases during the operation of the platoon.

### 2.6 Autonomous controller for string stable operation

This investigation explores the autonomous operation of electric trucks in a platoon setup. Such conditions necessitate a controller to uphold the desired speed and position for ensuring string stability [8], [4]. Under autonomous platoon operation, each truck's controller endeavours to match the speed of the lead vehicle while maintaining the desired spacing between vehicles. The controller should mitigate any spacing errors resulting from potential disturbances on the road, thereby ensuring string stability [8], [27] through appropriate drive or brake inputs.

Each electric truck in the platoon is equipped with an autonomous controller based on the Sliding Mode Control (SMC) strategy, ensuring string stability. The design process of this decentralized controller is outlined in detail in [15]. This controller determines the necessary drive or brake torque for each vehicle by considering the position and speed of the preceding vehicle. Its objective is to minimize tracking errors in speed and desired intervehicular distance, preventing their propagation along the platoon. To achieve string stability, the autonomous controller in each vehicle relies on speed and position data from the immediate preceding truck.

### 2.7 Energy consumption model

Equation (19) gives the total energy consumed ($E_{dc}$) as a function of the total power applied to the wheels ($P_{wheel}(t)$) as,

(19) $$E_{dc}=\int_{0}^{t_{dc}}P_{wheel}(t)dt.$$

In the above equation, $t_{dc}$ indicates the travel duration. The aggregate power applied to the wheels encompasses both the power needed to counteract resistive forces and inertial forces. For an electric truck, this constitutes the entirety of power required by the electric motors which can be expressed in equation (20) as

(20) $$P_{wheel}(t)=P_{mot}(t)=F_{R}(t)v(t)+ma(t)v(t).$$

At lower speeds, a greater amount of energy is required to counteract inertia and/or grade resistance. However, as the speed of operation rises, the significance of aerodynamic drag increases. Consequently, during high-speed truck travel, the primary energy consumption is dedicated to overcoming aerodynamic drag.

This force for each truck in the platoon is denoted in equation (21). It can be expressed as

(21) $$F_{a}(t)=\rho \;a_{f}\;C_{D}(t)\frac{v{\left(t\right)}^{2}}{2}.$$

Equation (22) gives the drag coefficient $C_{D}(t)$ [28]. It can be expressed as

(22) $$C_{D}(t)=C_{D0}(\gamma_{1}d_{i}{\left(t\right)}^{\gamma_{2}}+\gamma_{3}).$$

In this context, $C_{D0}$ denotes the drag coefficient of an individual vehicle (not operating within a platoon formation), with the parameters $\gamma_{1}$, $\gamma_{2}$, and $\gamma_{3}$ empirically derived [28]. Equations (21) and (22) illustrate that reducing the intervehicular distance, $d_{i}(t)$, can lower the drag coefficient and consequently decrease aerodynamic drag force. This analysis is extensively discussed in [8]. A reduction in aerodynamic drag force results in diminished resistive forces ($F_{R}(t)$) and subsequently reduced $P_{mot}(t)$, leading to decreased energy demand from the battery (as described in equation (12)). Consequently, this extension in the range of electric truck operation is achieved. However, denser formations may elevate the risk of platoon instability and vehicle collisions.

## 3 Long short term memory (LSTM) network

**Figure 2:**
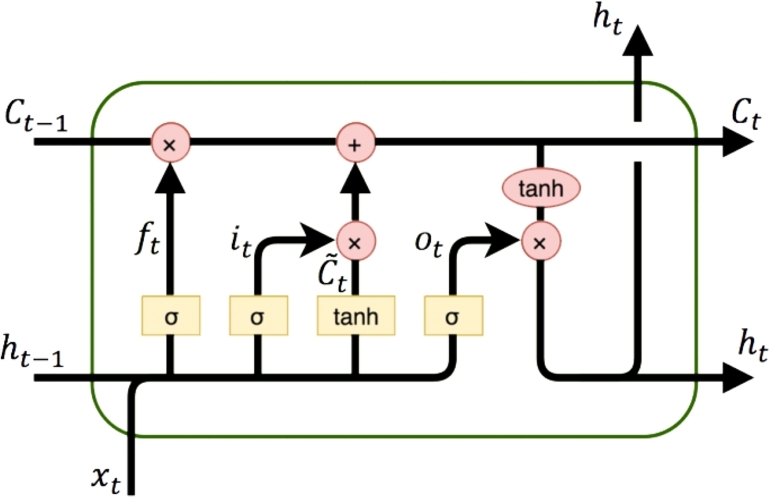
Structure of an LSTM layer [30].

A Recurrent Neural Network (RNN) is a type of neural network that can process sequential data by retaining information from previous inputs. LSTM is a type of RNN architecture that is designed to overcome the problem of vanishing gradients in traditional RNNs [29]. The vanishing gradient problem occurs when the gradients of the weights in the network become extremely small as they are propagated back through time, making it difficult for the network to learn long-term dependencies. Fig. 2 and Algorithm 1 present the layout and detailed steps involved in building an LSTM layer.

**Algorithm 1:**
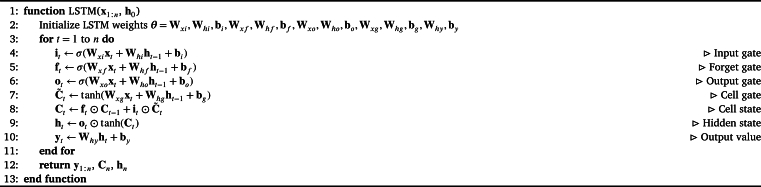
LSTM Network.

In this algorithm, the forward pass of a Long Short-Term Memory (LSTM) network is presented, which is a type of recurrent neural network (RNN) architecture. Given an input sequence $x_{1:n}$ and an initial hidden state $h_{0}$, the LSTM computes a sequence of hidden states $h_{1:n}$ and output values $y_{1:n}$.

The LSTM maintains a cell state vector $C_{t}$ that can store information over long periods of time. At each time step *t*, the LSTM computes four gates that control how information is processed and propagated through the network. The input gate $i_{t}$ determines how much of the input at time *t* is added to the cell state, the forget gate $f_{t}$ determines how much of the previous cell state should be retained, and the output gate $o_{t}$ determines how much of the current cell state should be output as the hidden state $h_{t}$. The cell gate ${\overset{˜}{C}}_{t}$ determines how much of the input at time *t* should be added to the cell state.

Prior to the forward pass of the LSTM network, the model parameters need to be initialized. The parameters consist of weight matrices and bias vectors that are used to transform the input and hidden state at each time step. Specifically, the weight matrices include $W_{xi}$, $W_{hi}$, $W_{xf}$, $W_{hf}$, $W_{xo}$, $W_{ho}$, $W_{xg}$, $W_{hg}$, and $W_{hy}$, while the bias vectors are $b_{i}$, $b_{f}$, $b_{o}$, $b_{g}$, and $b_{y}$. Together, these parameters form a set denoted as ***θ***. The initialization step sets the initial values of all parameters in ***θ***.

At each time step *t*, the LSTM computes the input gate $i_{t}$, forget gate $f_{t}$, output gate $o_{t}$, and cell gate $\overset{˜}{C}t$ using a sigmoid activation function *σ* and the input at time *t* and the previous hidden state $h_{t-1}$. The cell state $C_{t}$ is updated using the input gate $i_{t}$, forget gate $f_{t}$, and cell gate ${\overset{˜}{C}}_{t}$. The hidden state $h_{t}$ is computed using the output gate $o_{t}$ and the updated cell state $C_{t}$. Finally, the output value $y_{t}$ is computed as a linear function of the hidden state $h_{t}$. The algorithm returns the sequence of output values $y_{1:n}$, the final cell state $C_{n}$, and the final hidden state $h_{n}$.

Sequence-to-sequence (Seq2Seq) networks are a type of deep learning model that can process sequences of variable length and generate output sequences of variable length. They are often used for tasks such as machine translation, speech recognition, and text summarization, and could be potentially adaptable for the current task i.e., speed planning for vehicle platooning. The next section describes a type of Seq2Seq network, called the encoder-decoder LSTM network, which has been appropriately adopted for electric truck platoon speed prediction.

## 4 Encoder decoder LSTM network

**Figure 3:**
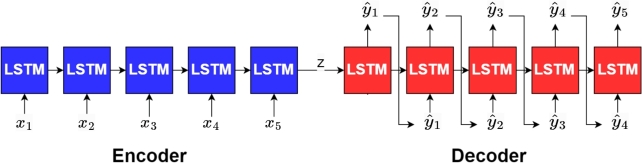
Encoder decoder LSTM model.

An encoder-decoder LSTM network is a specific type of encoder-decoder architecture that uses LSTM units to capture temporal dependencies in sequential data, such as natural language or time-series data. This architecture consists of an encoder LSTM network that transforms the input sequence into a fixed-length vector representation and a decoder LSTM network that generates the output sequence (Fig. 3).

One of the key advantages of these networks is their ability to handle variable-length input and output sequences, which is important for tasks such as machine translation where the input and output sequences can have different lengths in different languages. Additionally, they can capture long-term dependencies in the input sequence which allows them to generate more accurate and coherent output sequences. They are powerful and flexible approaches to sequence modelling that have been successfully applied to a wide range of natural language processing tasks.

In this study, a sequential data model that is suitable for predicting the speed profile of a leader vehicle in the platoon system based on the instantaneous power input and the operating conditions is presented. Let P(t) be the power at any time instant *t*. Every drive cycle was divided into groups of an arbitrary number *N*. If the total instances were not divisible by the group size, zero padding was applied before the first group. For example, the validation drive cycle has 208300 instances. For N=500, 200 instances of 0 were prefixed to this drive cycle so that it becomes divisible by 500.

Consider an arbitrary group *j* of *N* instances ${\overset{ˆ}{p}}_{j}=[p(t),p(t+\Delta t),...,p(t+(N-1)\Delta t)]$ where, Δ*t* is the sampling time i.e., 0.01 s. The initial few observations of each group will be missing significant information and dependency from previous instances as they are in the previous group. To accommodate this, every input sequence will be prefixed with an arbitrary number (*M*) of instances from the previous group (from the end). The final input sequence was then $p_{j}=[p(t-M\Delta t),....,p(t-\Delta t),p(t),p(t+\Delta t),...,p(t+(N-1)\Delta t)]$ with length (M+N). This sequence $p_{j}$ is given as input to the encoder LSTM. This generates a hidden contextual representation of the power sequence. Mathematically, the encoder LSTM can be represented as follows (Equation (23)):

(23) $$h_{et},C_{et}={\text{LSTM}}_{\text{encoder}}(p(t),h_{e(t-1)},C_{e(t-1)})\;\text{where }t\in [t-M\Delta t,t+(N-1)\Delta t],$$

where, $h_{et}$ is the hidden state of the encoder LSTM at time *t*, $c_{et}$ is the cell state of the encoder LSTM at time *t*, and ${\text{LSTM}}_{\text{encoder}}$ is the LSTM function with trainable parameters $\theta_{\text{e}}$. The initial hidden state $h_{e(t-(M-1)\Delta t)}$ and cell state $C_{e(t-(M-1)\Delta t)}$ are both set to zero.

The final hidden state $h_{e(t+(N-1)\Delta t)}$ represents the fixed-length vector representation of the input sequence, which contains the most important information necessary for generating the output. However, the operating conditions have not been incorporated yet. To accommodate the same, the output of the encoder LSTM (which encompasses the hidden representations) was combined with the operating conditions and passed into the decoder LSTM. The encoder LSTM's final hidden state is given as $h_{e(t+(N-1)\Delta t)}$ and the cell state is given by $C_{e(t+(N-1)\Delta t)}$. The next step is to include the operating conditions and pass it as initial decoder input along with the hidden representations. Equations (24) and (25) give the expression for the initial decoder input which can be represented as $\left[h_{d0},C_{d0}\right]$. The equation is given by

(24) $$h_{d0}=[h_{e(t+(N-1)\Delta t)},\mu_{j},H_{j},m_{j}],$$

(25) $$C_{d0}=[C_{e(t+(N-1)\Delta t)},\mu_{j},H_{j},m_{j}].$$

Here, $\mu_{j},H_{j},m_{j}$ are the friction coefficient, time-headway and mass respectively of that corresponding group.

An iteration of the decoder LSTM can be represented as follows (Equation (26)):

(26) $${\overset{ˆ}{y}}_{j}(i),h_{di},C_{di}={\text{LSTM}}_{\text{decoder}}({\overset{ˆ}{y}}_{j}(i-1),h_{d(i-1)},C_{d(i-1)}),$$

where, ${\overset{ˆ}{y}}_{j}(i)$ is the $i^{th}$ decoder output of this group j, ${\overset{ˆ}{y}}_{j}(i-1)$ is the output at the previous instant, $h_{di}$ and $C_{di}$ are the hidden state and cell state of the decoder LSTM, ${\text{LSTM}}_{\text{decoder}}$ is the LSTM function with trainable parameters $\theta_{\text{d}}$.

The decoder LSTM generates an output of a size equal to the length of the hidden representations. Hence, it was passed into a fully connected linear layer for the final output to match the dimensions of the input group i.e., *N*, which essentially is the speed of the leader vehicle at the same instances of that particular input group (excluding the previous instances included).

If $\overset{ˆ}{v}(t)$ represents the predicted speed of the truck at an instant *t*, the final output ${\overset{ˆ}{v}}_{j}=[\overset{ˆ}{v}(t),.....,\overset{ˆ}{v}(t+(N-1)\Delta t)]$, where, *j* is the index of that particular group (input is $p_{j}$) is given by (Equation (27))

(27) $${\overset{ˆ}{v}}_{j}=W_{v}{\overset{ˆ}{y}}_{j}+b_{v},$$

where ${\overset{ˆ}{y}}_{j}$ is the vector representing the decoder output of this group. The complete model structure of the above-discussed encoder-decoder LSTM for platoon speed prediction is given in Algorithm 2.

**Algorithm 2:**
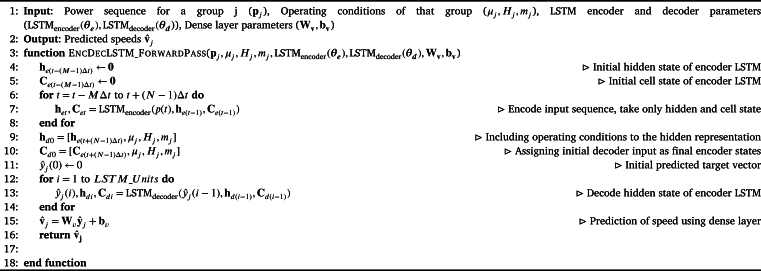
Encoder-Decoder LSTM model structure.

### 4.1 Training and validation

The previous subsection described the structure of the encoder-decoder LSTM model used in this study. This section describes the steps involved in training and validation, along with the metrics used to compare the model while hyperparameter tuning.

Feature scaling is an important pre-processing step for many machine learning algorithms [31]. If features are left unscaled, there is a high chance that some features dominate due to their sheer order of magnitude and create an unnecessary bias in the model. Since the range and scale of each variable is different in the considered data set, all the variables were normalized to lie in between 0 and 1.

Training is performed by backpropagation using Gradient Descent which involves computing the gradients with all the hyperparameters [32]. The concise list of all the hyperparameters involved with this model is shown in Table 1.

**Table 1:** Hyperparmeter description.

Hyperparameter name/symbol	Description
*M*	Number of instances from previous group prefixed to the current group as input
*N*	Number of instances taken per group for an input sequence
*learning_rate*	Learning rate in the gradient update equation
*LSTM_Layers*	Number of LSTM layers in encoder and the decoder
*LSTM_Units*	Dimensionality of the LSTM output space i.e., hidden representation z
*epochs*	Number of iterations
*batch_size*	Number of sequences processed before updating the weights

Training is followed by validation. For validating a model, appropriate performance metrics and data have to be defined. Since this is a regression problem (the quantity predicted is speed which is a continuous variable), Root Mean Squared Error (RMSE) (Equation (28)) was chosen as the performance metric. Moreover, Mean Absolute Percentage Error (MAPE) (Equation (29)) was also used as a performance metric to compare the predicted output with the actual drive cycle as a percentage change. Mathematically, they are defined as

(28) $$\mathrm{RMSE}=\sqrt{\frac{1}{n}\sum_{i=1}^{n}{\left(v_{i}-\overset{ˆ}{v_{i}}\right)}^{2}},$$

(29) $$\mathrm{MAPE}=\frac{100\text{\%}}{n}\sum_{i=1}^{n}\left|\frac{v_{i}-{\overset{ˆ}{v}}_{i}}{v_{i}}\right|.$$

If the performance of the training data is only evaluated, it would result in overfitting, i.e., the model may become too specialized to the training data and fail to generalize to new, unseen data. To address this issue, the available dataset is typically split into training and validation sets. The model is trained on the training set, and its performance is evaluated on the validation set, which serves as a proxy for the unseen test data. This allows us to monitor the model's performance on new data and detect if it is overfitting to the training data. Therefore, validation data is not part of the training data to ensure that the model is evaluated on data that it has not encountered during training and to prevent overfitting.

## 5 Data collection procedure

The process of developing a deep learning model requires a data set of known inputs and outputs for training and validation. Using the electric truck platoon framework [8], SOC profiles for different drive cycles for different operating conditions could be obtained. These SOC profiles were used as the data set to train and validate the model.

**Figure 4:**
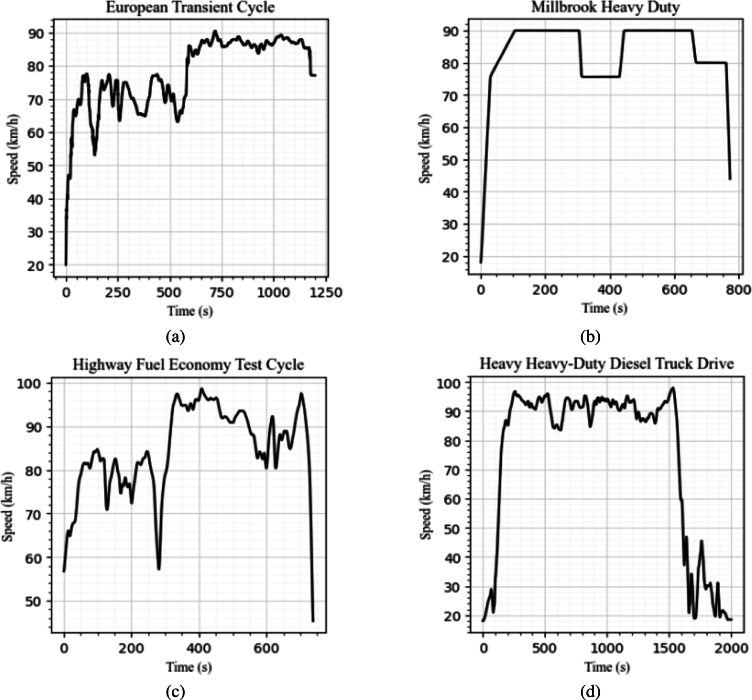
Drive cycles used, (a) European Transit Cycle, (b) Millbrook Heavy Duty, (c) Highway Fuel Economy Test Cycle, (d) Heavy Heavy-Duty Diesel Truck Drive.

In this work, four commonly used heavy vehicle highway drive cycles, the European Transient Cycle (ETC) [33], [34], Millbrook cycle [34], the Highway Fuel Economy Test (HWFET or HFET) cycle [34], [35], and the Heavy Heavy-Duty Diesel Truck (HHDDT) cycle [34], [36], were used. Their details are tabulated in Table 2, and plots are presented in Fig. 4.

• The ETC is used for emission certification of heavy-duty diesel engines in Europe starting in the year 2000 [33].
• Millbrook cycles [34] are an extensive list of test cycles developed by Millbrook Proving Ground, which is an English vehicle testing centre and is one of the largest vehicle testing centres in Europe. The heavy-duty motorway cycle was taken as one of the drive cycles for training this model.
• The HWFET cycle is a chassis dynamometer driving schedule developed by the US Environmental Protection Agency (EPA) [35]. The driving cycle is developed for simulating a mixture of interstate highway and rural driving.
• The HHDDT schedule [36] is a chassis dynamometer test developed by the California Air Resources Board with the cooperation of West Virginia University. The test consists of four speed-time modes, including idle, creep, transient and (high speed) cruise. As this study mainly focuses on highway operation, the cruise mode was taken as the drive cycle to be used to validate the deep learning model.

**Table 2:** Drive cycles used.

Purpose	Name of Cycle	Distance (m)	Duration (s)	Avg. Speed (km/h)
Training	European Transient Cycle - part 2 and 3	25620	1200	76.85
	Millbrook Heavy Duty - motorway	17903	780	82.6
	Highway Fuel Economy Test Cycle	16450	765	77.7

Validation	Heavy Heavy-Duty Diesel Truck Drive	37336	2083	64.21

For all these drive cycles, the SOC profiles were generated for various road and vehicle conditions using the developed electric platoon framework. Two different tire-road friction coefficients of values μ=0.3 and μ=0.8, representing low and high friction road conditions, respectively were considered. A platoon of homogeneous trucks under fully laden (16200 kg) and partially laden (10450 kg representing 60% loading) conditions were taken into account. Similarly, different time headway magnitudes (h=1 s and h=1.5 s), which is the time gap between two successive vehicles in the platoon, were also considered while generating the SOC profiles.

Using every possible combination of the above operating conditions, 8 SOC profiles were obtained, for each drive cycle, amounting to a total of 32 SOC profiles for the 4 considered drive cycles (24 training and 8 validation corresponding to the respective drive cycles as shown in Table 2).

The sampling time was taken as 0.01 seconds. Therefore, the total number of data points across the training data came out to be 21,96,000 and that of validation data is 16,66,400.

Since the goal is to predict the speed profiles according to the SOC discharge cycle, speed is considered as the target variable and other parameters, viz., time headway, road friction coefficient, vehicle mass, and battery SOC, represented as instantaneous power were selected as input features.

## 6 Model implementation

### 6.1 Baseline model

A baseline encoder-decoder LSTM model was initially created and used as a starting point for further improvements. The input sequence consisted of 100-time instances and was prefixed with the last 5 instances from the previous input sequence (*M* is 5 and *N* is 100). The implementation was carried out in Python®, utilizing the Keras [37] and TensorFlow [38] libraries. The default learning rate of 0.001 from the TensorFlow package was chosen for this model.

It is important to note that the selection of hyperparameters is typically determined by experimentation and tuning on a validation set. An approach to improve the baseline model can be to start with a small value for a hyperparameter and gradually increase it until the performance on the validation set stops improving or begins to degrade. Based on this strategy, a single LSTM layer was selected for both the encoder and decoder, with each layer containing 64 units. The model was trained for 20 epochs with a batch size of 8.

Since the aerodynamic drag is proportional to the square of the vehicle speed, the performance of a platoon is most effective and efficient only at higher speeds. Hence, it is reasonable to assess the performance of the platoon only during the high-speed portions. Considering this fact, the performance metrics were calculated only from 250 s to 1500 s in the validation dataset where the platoon is in high-speed operation. The RMSE was computed and found to be 12.62 km/h. The MAPE was obtained to be 13.25%. This value served as the benchmark for further improvements through hyperparameter tuning, which is discussed in the subsequent subsection.

### 6.2 Hyperparameter tuning and final model

Hyperparameter tuning is a crucial step in deep learning that involves finding the optimal values of the hyperparameters that govern the behaviour of the model. It helps in improving model performance, reducing overfitting, and ensuring faster convergence. It is important to experiment with different hyperparameters and evaluate their impact on the model's performance to find the optimal values that best suit the specific problem at hand. The range of values for each hyperparameter is listed as shown in the second column in Table 3.

**Table 3:** Hyperparameter tuning and the final model (Parameters are defined in Section 4 and Table 1).

Hyperparameter	Range of values used for tuning	Parameter value in final model
*M*	1, 5, 10, 50, 100, 500	5
*N*	10, 50, 100, 500, 1000, 5000	500
*learning_rate*	0.00001, 0.001, 0.01, 0.1	0.001
*LSTM_Layers*	1, 2, 3	1
*LSTM_Units*	64, 128, 256, 512, 1024	256
*epochs*	10, 20, 50, 100	10
*batch_size*	8, 16, 32, 64	8

From the baseline model with the values of *M* and *N* respectively being 5 and 100, different combinations of *M* and *N* were tested in both directions (higher and lower). The best model resulted when *M* is 5 and *N* is 500.

The learning rate is an important hyperparameter to tune because it affects the convergence speed and accuracy of the model. The learning rate trade-off refers to the balance between the convergence speed and the accuracy of the model during the training process. In general, a high learning rate leads to faster convergence but may cause the optimization algorithm to overshoot the optimal solution and lead to oscillations or even divergence. On the other hand, a low learning rate leads to a more accurate solution, but it may take a longer time to converge and can be sensitive to initialization. Hence, a range of values from 0.00001 to 0.1 was considered and the best model had a learning rate of 0.001.

In general, the number of units of the LSTM layer determines the number of memory cells in the layer, which enables the model to capture long-term dependencies in the input sequence. A larger value can potentially improve the model's capacity to learn complex patterns in the data but also requires more computational resources and may lead to overfitting if the training data is limited. Considering this, values from 64 to 1024 were chosen for tuning and the model with 256 units gave the best performance.

The number of LSTM layers was tuned in increments of 1, starting from a single layer. It was observed that as the number of layers increased, there was no improvement in performance. Hence, one layer each for the encoder and decoder was chosen.

Following a similar strategy for the other hyperparameters (epochs and batch_size), the model with the most optimum performance metric was chosen. The final model bears the hyperparameters shown in the last column of Table 3. The RMSE on validation data for this model was obtained to be 3.57 km/h, which is the best among all the models trained. Concurring with this, the MAPE for this model was obtained to be 2.94%, the lowest among the trained models. Hence, this was finalized as the best model.

**Figure 5:**
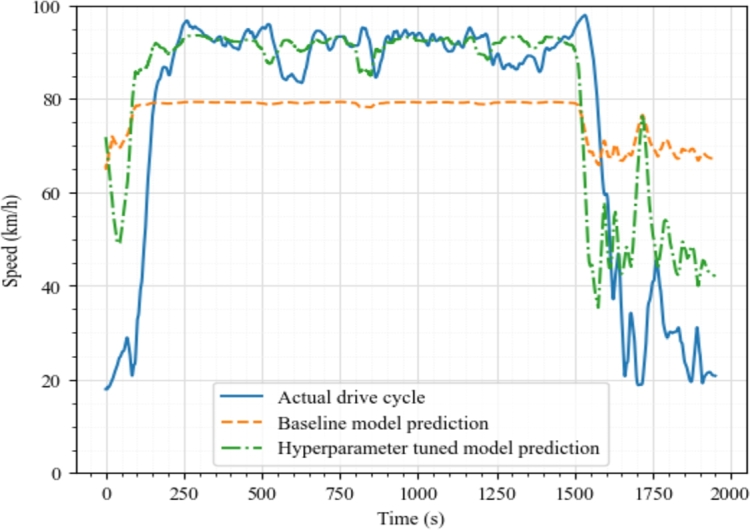
Comparison between baseline model and final model predictions for the case *μ* = 0.3,*h* = 1,*m* = 16200.

Fig. 5 shows the predictions of the baseline model and the final model on the validation data along with the actual drive cycle for μ=0.3, h=1 s, m=16200 kg. From the plot, it can be observed that hyperparameter tuning improved the performance of the model and the final model fits better than the baseline model, which was underfitting on the validation dataset.

The models were run in a PC that uses a 1.6 GHz 8th Generation Intel i5 Processor, with 12 GB installed RAM. No GPU was used for running. The training time for this current dataset and the final model hyperparameters came out to be 11 minutes. Validation and implementing this model took 12 seconds. This model can be used as a benchmark for future developments. Expanding computational capabilities and improving efficiency is deliberated upon to be one of the areas of future work.

## 7 Results and discussions

**Figure 6:**
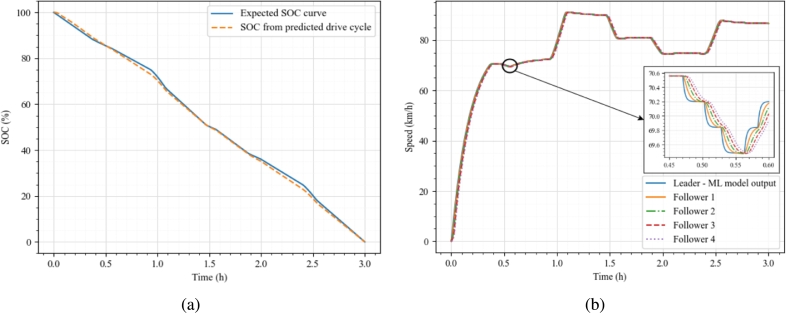
Drive cycle prediction (a) SOC curve, (b) Predicted drive cycle and platoon tracking profiles.

The proposed deep learning-based framework could predict the electric truck platoon's speed profiles/drive cycles to suit a particular driving condition, route and operating conditions based on a particular energy demand profile. Such a profile could be generated, considering multiple factors like prior route information, vehicle and battery capacity, and availability of charging infrastructure. For instance, an arbitrary battery usage profile as shown in Fig. 6(a) (presented in terms of SOC curve) was generated for a fully laden electric truck (m=16200 kg) equipped with a battery pack capacity of 270 kWh operating on a dry level road. All the electric truck parameters as presented in [15] have been used to simulate the vehicles. The battery pack was assumed to be able to satisfy the power demanded by the electric motor irrespective of the SOC magnitude, and the vehicle was driven continuously till the whole battery was exhausted (which is ≈ 3 h). This should give an approximate range of 220 - 280 km based on various road conditions.

Fig. 6(b) presents the predicted speed profile corresponding to an individual truck's energy demand as given by Fig. 6(a). The platoon is then made to follow the predicted/generated speed profile, and the tracking plots are presented in Fig. 6(b). The autonomous controller ensured stable platoon operation and the follower trucks were able to follow the predicted drive cycle without collisions. The platoon could travel ≈240 km for the presented full discharge cycle. The actual SOC profile corresponding to the leader vehicle obtained from the controller framework is also presented (dashed curve in Fig. 6(a)), which closely follows the demanded SOC curve. These results show the capability of the framework in predicting the drive cycles for the electric truck platoon such that the platoon is string stable and each truck in the platoon is able to track the available SOC profile to meet battery constraints.

**Figure 7:**
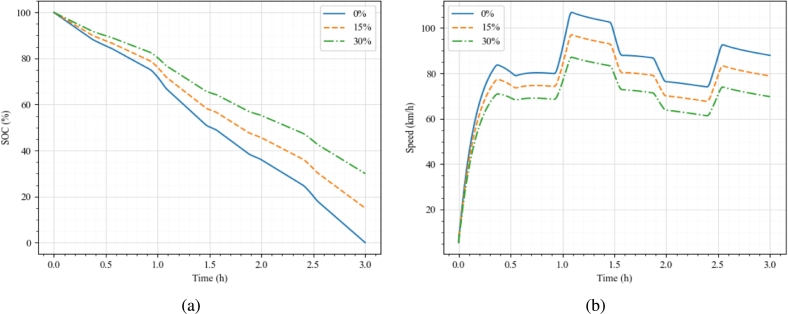
On-demand drive cycle prediction/adaptation (a) Demanded SOC profiles, (b) Predicted drive cycles.

The proposed framework could adapt the drive cycle predictions according to various demands posed by the user. For instance, ideally, it is not advised to drain the battery below a threshold magnitude (as presented in Fig. 6(a)), but to maintain/charge the battery once the SOC magnitude is below a threshold magnitude. Fig. 7(a) presents such scenarios where the minimum SOC magnitudes are chosen to be 0%, 15%, and 30% at the end of a three-hour continuous operation. The predicted drive cycle profiles to achieve these SOC profiles are presented in Fig. 7(b). All three cases are presented for the same road condition, vehicle mass and time headway magnitudes (μ=0.8, *m*= 16200 kg, *h* = 1 s). This was the reason to have a similar drive cycle profile in all three cases with different magnitudes. For minimum SOC magnitudes of 0%, 15%, and 30%, the maximum speed that the platoon could achieve during the cruise phase was found to be 110 km/h, 95 km/h, and 85 km/h, respectively, and minimum speed to be 75 km/h, 65 km/h, and 60 km/h, respectively. Travelling at a higher speed increases the aerodynamic drag force, thus increasing the energy consumption. However, by virtue of operating in a platoon formation, the aerodynamic drag force is comparatively lower than that of an individual vehicle, thus allowing the platoon to operate at higher speeds [8]. One should note that the drive cycles presented are not absolute, but a representative guideline for the vehicle to achieve the desired SOC at each instant. The instantaneous speed values predicted by the model could be interpreted as an “upper ceiling” of the operating speed while the instantaneous power demand does not exceed the desired magnitude.

### 7.1 Case study: electric truck platoon routing problem

**Figure 8:**
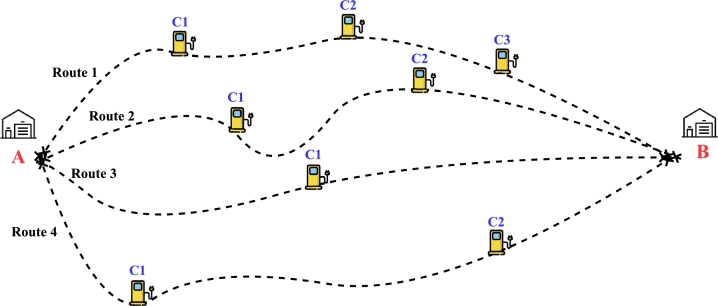
HGV platoon routing problem schematic (not to scale).

The presented framework could potentially be used to formulate policy decisions such as route selection between two locations based on multiple factors like road type, route information, and constraints such as road elevation, speed limitations, and availability of charging infrastructures. Fig. 8 presents such a routing problem where an electric truck platoon has been planned to travel between two warehouses ‘A’ and ‘B’ with options to travel 4 different routes, Route 1 to Route 4. Each route was assumed to be having different lengths, (with Route 1 being the longest and Route 3 being the shortest) and was equipped with charging stations at varying locations. Each vehicle in the platoon is assumed to be fully loaded with a battery pack capacity of 270 kWh with an approximate range of 220 - 280 km from a fully charged condition. Each charging station (as presented in Fig. 8) is assumed to be equipped with fast charging technology which could fully charge the batteries in ≈2 h. The speed profiles were predicted considering the time constraint, platoons taking any route from ‘A’ to ‘B’ should have similar travel time.

**Figure 9:**
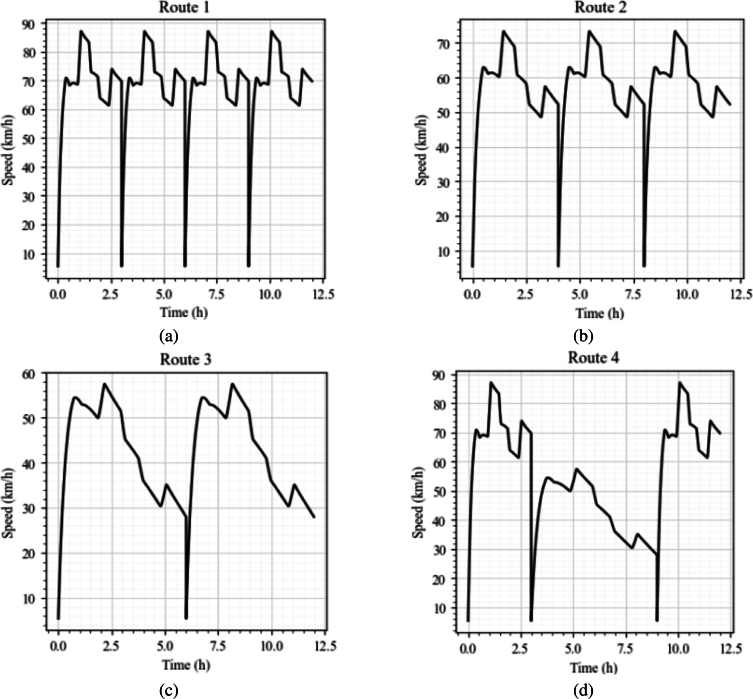
Predicted drive cycles for the different routes, (a) Route 1, (b) Route 2, (c) Route 3, (d) Route 4.

The framework predicts the speed corresponding to each route, and charging location, and time to reach the destination. For instance, while travelling through the longest route (Route 1) the platoon has to travel faster compared to other routes to reach the destination at the same time as other routes. Route 1 has three charging stations, where the batteries are fully charged and the platoon can resume its travel. The predicted speed profiles are presented in Fig. 9. The idling profiles during charging are not shown for brevity. Similarly, for Route 3, one can opt to drive at lower speeds. This is also necessary as the charger location is much farther, and one has to opt for lower speeds, and lower acceleration/deceleration rates to minimize the energy consumption and extend range. The model could predict the speed profiles adopting to the route requirements and constraints, giving the fleet operator to choose a route of their convenience and capabilities. One should note that the presented routing problem is not absolute, but just a sample case to show the capability of the proposed approach. The framework has the flexibility to adopt its parameters incorporating a wide range of operating conditions and constraints to give policy decisions for optimal electric truck routing.

In addition to predicting the drive cycles, the presented framework could also give different descriptive parameters representing the drive cycle called kinematic parameters [23], [34]. The parameters for the considered case are computed as proposed by [23] and are presented in Table 4. These kinematic parameters could give further insights into the driving patterns to be followed and the capabilities and constraints of individual vehicles in the platoon. These parameters could also be used as a quantitative measure to represent different road and traffic conditions [23]. Sixteen critical parameters characterising each predicted drive cycle (for each route) were computed and presented. These values are well within the practical limits and comparable with those of conventional drive cycles presented in [23]. The predicted drive cycles have average speeds of 69.1, 57.5, 42.7, and 55.9 km/h for Routes 1 - 4, respectively, with Route 1 being faster and Route 3 being slower with maximum speeds of 87.1 km/h, and 57.5 km/h, respectively. The acceleration and deceleration values have a direct impact on energy consumption. Considering Route 3 has the most distance between the charging points, the platoon has to consume less average power in this route compared to the other three routes. The average acceleration parameter in Table 4 corroborates this fact, where the value corresponding to Route 3 was approximately 2.86, 2.07, and 5.65 times smaller compared to that of Route 1, 2, and 3, respectively. Apart from using the kinematic parameters for drive cycle/driving profile characterisation, these parameters could give further insights into the route, charging location placement, required battery capacity to complete a particular route and further design strategies.

**Table 4:** Kinematic parameters for the predicted drive cycles.

Group	Parameter	Units	Route 1	Route 2	Route 3	Route 4
Distance related	Total distance	km	830	690.5	512.2	671.1

Time related	Total time	s	43200	43200	43200	43200
	Drive time spent accelerating	s	12592.8	11538.29	9939.024	11154.672
	Drive time spent decelerating	s	30607.09	31661.57	33260.76	31810.64
	% of time accelerating	%	29.15	26.71	23.00	25.82
	% of time decelerating	%	70.85	73.29	76.99	73.64

Speed related	Average speed	km/h	69.166	57.542	42.690	55.93
	Standard deviation of speed	km/h	11.758	9.869	10.323	16.84
	Maximum speed	km/h	87.137	73.405	57.485	87.137

Acceleration related	Average acceleration	m/s^2^	0.000414	0.000301	0.000145	0.000819
	Average positive acceleration	m/s^2^	0.00862	0.0057	0.00343	0.00642
	Average negative acceleration	m/s^2^	-0.00296	-0.0017	-0.00084	-0.0013

Stop related	Number of stops	-	4	3	2	3
	Number of stops per km	/km	0.00482	0.004345	0.003904	0.00447
	Average stop duration	s	7200	7200	7200	7200
	Average distance between stops	km	207.5	230.2	256.1	223.7

## 8 Conclusion

A systematic approach towards electric truck platoon route planning for long-haul freight movement has been addressed utilizing deep learning. Since the existing work on route planning based on the energy consumption criterion of platoons is limited and using the first principles approach is cumbersome, an analytical solution for a speed planner to predict the drive cycle for an electric truck platoon based on the desired/available battery SOC profile has been developed. An encoder-decoder LSTM model was used as both the input and the output data are sequential. One major limitation of this model is that the dataset should ideally be based on true data by physically running the platoons but using a controller might induce inaccuracies. However, since the data collection for the model development has been done using a detailed vehicle dynamics-based model of an electric truck platoon framework, the predicted drive cycles are expected to be realistic and hence can be relied upon for taking policy decisions for planning the route.

Moreover, the framework has been trained to incorporate a wide range of operating conditions such that the predicted drive cycles would not lead to string instability issues during autonomous platoon operation. Future work would include incorporating the models to subsequent follower vehicles and investigating other use cases in vehicle platooning where machine learning/deep learning can be applicable, along with accommodating factors such as road slope to make the model more complex and closer to reality. Another area of progress could be to investigate different models/approaches to this problem as the previously existing work is limited. The possibility to consider computational efficiency and the scope to expand the capabilities can also form a basis for further improvement. This study presented in this paper serves as a benchmark for comparison for any future work in this scope.

## CRediT authorship contribution statement

**S. Karthik:** Writing – original draft, Visualization, Validation, Software, Methodology, Investigation, Formal analysis, Conceptualization. **G. Rohith:** Writing – review & editing, Visualization, Supervision, Software, Investigation, Formal analysis, Conceptualization. **K.B. Devika:** Writing – review & editing, Supervision, Methodology, Funding acquisition, Conceptualization. **Shankar C. Subramanian:** Writing – review & editing, Visualization, Supervision, Resources, Funding acquisition.

## Declaration of Competing Interest

The authors declare that they have no known competing financial interests or personal relationships that could have appeared to influence the work reported in this paper.

## Data Availability

Data will be made available on request.
